# Short-term outcomes of low anterior resection with and without ileostomy for low, mid and upper rectal cancers

**DOI:** 10.1007/s13304-025-02088-2

**Published:** 2025-01-23

**Authors:** Vincent Xu, Kristina La, Rachel Ma, Paola Solis-Pazmino, Abbas Smiley, Moshe Barnajian, Joshua Ellenhorn, Roberto Bergamaschi, Yosef Nasseri

**Affiliations:** 1 The Surgery Group of Los Angeles, 8635 W 3Rd St, Suite 880, Los Angeles, CA 90048 USA; 2 Cedars Sinai Medical Center, Los Angeles, CA USA; 3 Surgery Department, Santa Casa de Porto Alegre, Porto Alegre, RS Brazil; 4 Knowledge and Evaluation Research Unit, Mayo Clinic, Rochester, MN USA; 5 CaTaLiNA-Cancer de Tiroides en Latino America, Quito, Ecuador; 6 Department of Surgery, Westchester Medical Center, Valhalla, NY USA; 7 Jacobi Medical Center, Department of Surgery, New York City Health Hospitals, New York, NY USA; 8 UT Southwestern Medical Center, Dallas, TX USA

**Keywords:** Elective low anterior resection, Diverting loop ileostomy, Rectal cancer, Short-term postoperative outcome, Anastomotic leakage, Tumor location

## Abstract

Although the addition of an ileostomy to low anterior resection (LAR) may often be considered preventative of anastomotic leakage (AL), evidence that clearly demonstrates such benefit is lacking. This study aimed to identify the impact of adding an ileostomy upon AL and organ-space surgical site infection (SSI) rates in patients with lower, middle, or upper rectal cancer. This case–control study included rectal cancer patients who had undergone elective LAR in the American College of Surgeons-National Surgical Quality Improvement Program dataset between 2016 and 2022. Patients with lower, middle, and upper tumors were identified and analyzed according to whether an ileostomy was added or not. Patients’ pre-, intra-, and short-term post-operative data were compared using univariable and multivariable methods. A total of 4048 patients (61.6% males) with a mean age of 60 years, whereof 1166 with lower, 1836 with middle, and 1046 with upper tumors were identified. An ileostomy was added in 2804 (69.3%) patients. Patients with upper tumors had an ileostomy added less frequently (78.5%vs 74.5% vs 49.9%, p < 0.001). The overall AL and organ-space SSI rates were 4.3% and 6.7%. There were no statistically significant differences in AL and organ-space SSI rates (requiring or not requiring re-intervention or re-operation) between patients with and without ileostomy regardless of tumor location. Multivariable logistic regression controlling for confounding variables showed no association between adding an ileostomy and AL and organ-space SSI rates (requiring or not requiring re-intervention or re-operation) regardless of tumor location. This case–control study did not find any evidence in support of a preventative impact upon AL and organ-space SSI rates of adding an ileostomy to LAR in patients with lower, middle, or upper rectal cancer.

**Supplementary Information:**

The online version contains supplementary material available at 10.1007/s13304-025-02088-2.

## Introduction

Various critical short-term postoperative complications can arise following a low anterior resection (LAR) of the rectum, with the most feared ones being anastomotic leakage (AL) and organ space surgical site infection (SSI). There are reported AL rates ranging from 3 to 23% following LAR [14]. A proximal fecal diversion via a loop ileostomy is often formed in hope of reducing AL and related postoperative complications post LAR. However, there are no recent high-level evidence or large database studies to demonstrate clear significant benefits of a diverting ileostomy in rectal cancer patients.

Studies have shown inconsistent results regarding the protective effects of an ileostomy procedure (regarding leak rate, mortality, morbidity, and other complications) [1–3, 5, 6, 10, 11]. Despite previous randomized controlled trials [7, 10, 12] and meta-analyses supporting the effectiveness of an ileostomy in reducing AL, several recent studies have shown that having an ileostomy following LAR does not improve AL rates [8, 11, 13]. Furthermore, studies comparing the outcomes following LAR with and without an ileostomy for different rectal cancer locations (high, mid and low rectum) are lacking. Therefore, we saw the need to update and carefully assess the effects of having a concurrent ileostomy on the short-term postoperative outcomes following LAR.

We used a large national database to identify the impact of adding an ileostomy upon AL and organ space SSI rates in patients with lower, middle, and upper rectal cancer.

## Methods

### Data and population

**Fig. 1:**
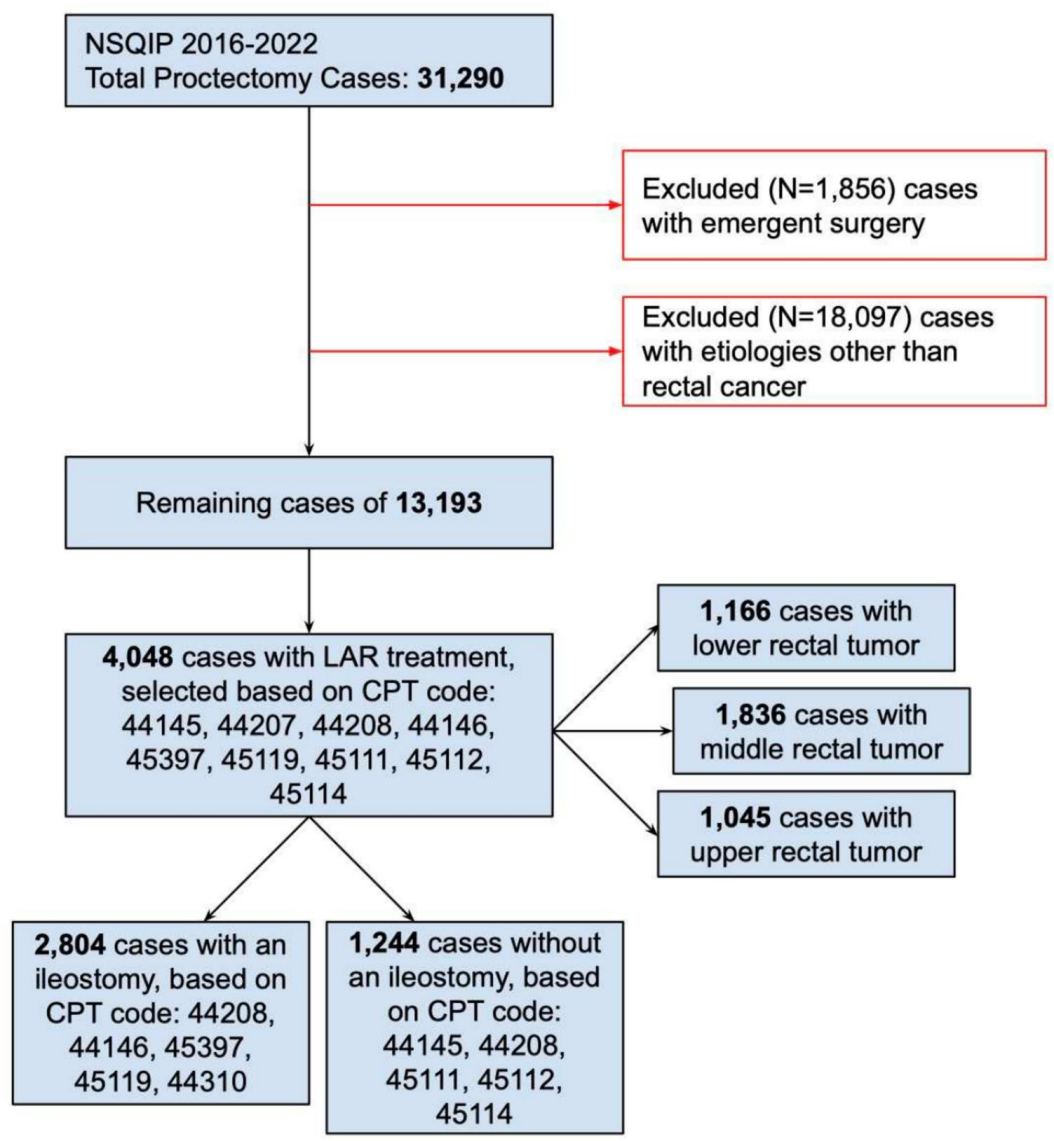
NSQIP database case selection criteria and flowchart

This case–control study included rectal cancer patients who had undergone elective LAR. Data were obtained from the American College of Surgeons National Surgical Quality Improvement Program (ACS NSQIP) Public Usage Files from 2016 to 2022 [15]. Patients undergoing proctectomy were further selected based on primary diagnosis of rectal cancer and the *International Classification of Diseases, 9th edition, Clinical Modification* (ICD9-CM) diagnosis code 154.1. Patients who underwent LAR were selected using Current Procedural Terminology (CPT) codes (CPT: 44,145, 44,207, 44,208, 44,146, 45,397, 45,119, 45,111, 45,112, 45,114). Within the cohort, patients undergoing a diverting loop ileostomy (referred as ileostomy) were identified using CPT codes of 44,208, 44,146, 45,397, 45,119, 44,310. The location of the rectal tumor was indicated in the database as lower, middle, and upper. Only patients undergoing elective surgery were included for this analysis (see Fig. 1 for selection logistics).

### Covariates and outcomes

Covariates included preoperative patient characteristics (age, sex, BMI, etc.), comorbidities (dyspnea, diabetes, etc.), preoperative chemotherapy and radiation therapy, intraoperative characteristics (operation time, operational approach, blood transfusion, etc.).

We assessed the incidence of AL and organ space SSI for rectal cancer patients who had proctectomy with LAR procedure. We further subdivided AL into mild and severe cases. Mild AL refers to cases where no interventions or reoperations were necessary in addressing the AL. Severe AL refers to cases where the AL had to be addressed with either a re-intervention (i.e. radiographic guided drainage) or a reoperation. We also further broke down and compared cases with space organ infection that required a reoperation. We ultimately conducted multivariable logistic regression analysis to identify risk factors for AL and organ space SSI, with further sub-analysis of different tumor locations and with and without a diverting ileostomy.

### Statistical analysis

Descriptive statistics were used to summarize patient cohort characteristics. The entire patient cohort was divided into three separate cohorts based on tumor location (upper, middle, lower) for downstream analysis. Continuous variables were compared using ANOVA tests and reported with average for each cohort. Chi-square tests and univariate analysis were used to compare categorical variables. Multivariable logistic regression analysis with backward elimination was used to evaluate the association between an ileostomy and various short-term postoperative outcomes reported as odds ratio (OR) with a 95% confidence interval (CI). All statistical analyses were performed using SPSS v.22 (IBM, Rochester, NY). A p-value less than 0.05 was considered significant.

## Results

The present study cohort included a total of 4048 patients (61.6% males) with a mean age of 60, and a mean BMI of 27.81 kg/m^2^. The cohort consisted of 1166, 1836, and 1046 patients with tumors in the lower, middle, and upper third of the rectum, respectively. Patients with low rectal cancer had lower mean age (58.90 vs 60.23 vs 61.31, p < 0.001), lower mean BMI (27.49 vs 27.76 vs 28.23 kg/m2, p = 0.026), and lower rate of dyspnea (26.6% vs 30.6% vs 34.2%, p < 0.001) compared to patients with middle and upper rectal cancers. Patients with upper rectal cancer had lower rates of preoperative chemotherapy (34.9% vs 54.4% vs 56.0%, p < 0.001) and radiation (30.2% vs 51.0% vs 50.9%, p < 0.001) as compared to middle and lower cohorts (Table 1,2).

**Table 1:** Demographic and preoperative characteristics

Variables (%)	Total (n = 4048)	Lower* (n = 1166)	Middle** (n = 1836)	Upper*** (n = 1046)	p-value
Mean age	60.31 (12.50)	58.90 (12.76)	60.23 (12.31)	61.31 (12.46)	** < .001**
Sex					0.352
Female	402 (38.4)	467 (40.1)	687 (37.4)	402 (38.4)	
Male	644 (61.6)	699 (59.9)	1149 (62.6)	644 (61.6)	
Race/ethnicity					** < .001**
African American	198 (4.9)	63 (5.4)	95 (5.2)	40 (3.8)	
Asian	564 (13.9)	130 (11.1)	223 (12.1)	211 (20.2)	
White	2177 (53.8)	674 (57.8)	970 (52.8)	533 (51.0)	
Hispanic	188 (4.6)	82 (7)	66 (3.6)	40 (3.8)	
other	36 (0.9)	13 (1.1)	13 (0.7)	10 (1.0)	
unknown	1073 (26.5)	286 (24.5)	535 (29.1)	252 (24.1)	
ASA classification					**0.036**
> = 3	2398 (59.2)	687(58.9)	1119 (60.9)	592 (56.6)	
1 or 2	1646 (40.7)	479(41.1)	716 (39.0)	451 (43.1)	
Mean BMI (kg/m2)	27.81 (6.43)	27.49(6.05)	27.76 (6.37)	28.23 (6.93)	**0.026**
Current Smoker	608 (15.0)	174 (14.9)	285 (15.5)	149 (14.2)	0.649
Functional Status					0.138
Dependent	36 (0.9)	4 (0.3)	19 (1.0)	13 (1.2)	
Independent	4009 (99.0)	1161 (99.6)	1815 (98.9)	1033 (98.8)	
Dyspnea	1230 (30.4)	310 (26.6)	562 (30.6)	358 (34.2)	** < .001**
COPD	92 (2.3)	18 (1.5)	45 (2.5)	29 (2.8)	0.121
CHF	19 (0.5)	5 (0.4)	7 (0.4)	7 (0.7)	0.538
Steroid	93 (2.3)	29 (2.5)	43 (2.3)	21 (2.0)	0.743
Hypertension	1594 (39.4)	437 (37.5)	717 (39.1)	440 (42.1)	0.082
Disseminated Cancer	312 (7.7)	83 (7.1)	140 (7.6)	89 (8.5)	0.465
Bleeding Disorder	15 (1.4)	16 (1.4)	21 (1.1)	15 (1.4)	0.829
Systemic Sepsis	27 (0.7)	4 (0.3)	11 (0.6)	12 (1.1)	0.06
Wound Infection	8 (0.2)	1 (0.1)	5 (0.3)	2 (0.2)	0.045
Diabete	596 (14.7)	160 (13.7)	272 (14.8)	164 (15.7)	0.427
Cardiac Condition	38 (0.9)	12 (1.0)	19 (1.0)	7 (0.7)	0.576
Pretreatment Clinical Staging					** < .001**
T0, Tis	15 (0.4)	5 (0.4)	4 (0.2)	6 (0.6)	
T1-3	2770 (68.4)	849 (72.8)	1384 (75.4)	537 (51.3)	
T4, T4a-b	333 (8.2)	90 (7.7)	144 (7.8)	99 (9.5)	
Pathological Staging					** < .001**
T0, Tis	456 (11.3)	156 (13.4)	206 (11.2)	94 (9.0)	
T1-3	3054 (75.4)	858 (73.6)	1437 (78.3)	759 (72.6)	
T4, T4a-b	215 (5.3)	48 (4.1)	65 (3.5)	102 (9.8)	

Bold p-values are < 0.05, indicating statistically significant difference between the cohorts in comparison. Continuous variables and categorical variables were analyzed with univariate analysis and ANOVA tests respectively (proportions and standard deviations were reported in the bracket)

*Lower rectal cancer (< 5 cm from anal verge)

**Middle rectal cancer (5–10 cm from anal verge)

***Upper rectal cancer (> 10 cm from anal verge)

**Table 2:** Intraoperative characteristics and short-term postoperative outcomes for patients with different tumor locations

Variables (%)	Total (n = 4048)	Lower* (n = 1166)	Middle** (n = 1836)	Upper*** (n = 1046)	p-value
Preoperative chemotherapy	2016 (49.8)	653 (56.0)	998 (54.4)	365(34.9)	** < .001**
Preoperative radiation Therapy	1845(45.6)	593(50.9)	936(51.0)	316(30.2)	** < .001**
Operation time (Minutes)	294.48(121.52)	319.08(129.96)	293.13(120.26)	269.41(107.95)	** < .001**
Intraoperative transfusion	206 (5.1)	64 (5.5)	88 (4.8)	54 (5.2)	0.694
Operative approach					** < .001**
Open	1154 (28.5)	280 (24.0)	553 (30.1)	321 (30.7)	
Laparoscopy	1232 (30.4)	350 (30.0)	537 (29.2)	345 (33.0)	
Robotic	744 (18.4)	273 (23.4)	338 (18.4)	133 (12.7)	
Diverting ileostomy	2804 (69.3)	915 (78.5)	1367 (74.5)	522 (49.9)	** < .001**
Total length of stay (days)	5.40 (11.70)	5.40 (11.16)	5.76 (10.41)	4.76 (14.15)	0.088
Reoperation	188 (4.6)	58 (5.0)	87 (4.7)	43 (4.1)	0.608
Organ space SSI	272 (6.7)	73 (6.3)	136 (7.4)	63 (6.0)	0.274
Organ space SSI w/ Reoperation	87(2.1)	23(2.0)	37 (2.0)	27 (2.6)	0.533
Anastomotic leakage	176 (4.3)	46 (3.9)	84 (4.6)	46 (4.4)	0.709
Anastomotic leakage Severity					0.172
Severe	133 (3.3)	30 (2.6)	63 (3.4)	40 (3.8)	
Mild	43 (1.1)	16 (1.4)	21 (1.1)	6 (0.6)	

Bold p-values are < 0.05, indicating statistically significant difference between the cohorts in comparison. Continuous variables and categorical variables were analyzed with univariate analysis and ANOVA tests respectively (proportions and standard deviations were reported in the bracket)

*Lower rectal cancer (< 5 cm from anal verge)

**Middle rectal cancer (5–10 cm from anal verge)

***Upper rectal cancer (> 10 cm from anal verge)

Within the entire cohort, 2,804 patients (69.3%) had surgery with an ileostomy. Patients with tumors in the upper third of the rectum had a much lower rate of having a concurrent ileostomy compared to middle and lower cohorts (49.9% vs 74.5% vs 78.5%, p < 0.001). When comparing intraoperative characteristics of the three patient cohorts, patients with lower rectal cancer had longer operative time (319.08 vs 293.13 vs 269.41 min, p < 0.001), lower rate of open surgery (24.0% vs 30.1% vs 30.7%, p < 0.001), and a higher rate of robotic surgery (23.4% vs 18.4% vs 12.7%, p < 0.001) compared to middle and upper rectal cancer cohorts (Table 2).

Organ space SSI and AL were the postoperative outcomes. For the entire cohort, the organ space SSI and AL rates were 6.7% and 4.3%, respectively. Additionally, to assess the incidence rate of organ space SSI and AL with different severity, we focused on the rates of severe AL and patients with organ space SSI who also had reoperation. For the entire cohort, the overall rate of having organ space SSI with reoperation was 2.1% and severe AL rate was 3.3%.

There was no statistically significant difference in rates of organ space SSI (6.3% vs 7.4% vs 6.0%; p = 0.274) or AL (3.9% vs 4.6% vs 4.4%; p = 0.709) among low, middle, and upper rectal cancer cohorts. There was also no statistically significant difference among the three cohorts in organ space SSI with reoperation (2.0% vs 2.0% vs 2.6%; p = 0.533) and rates of severe AL (2.6% vs 3.4% vs 3.8%; p = 0.172).

A sub-analysis comparing postoperative outcomes in patients with and without an ileostomy was performed. In the overall cohort, patients who had an ileostomy had longer mean operative time (303.51 vs 274.11 min, p < 0.001) and lower rate of having intraoperative transfusion (4.4% vs 6.7%, p = 0.002). Patients with diverting ileostomy were more likely to undergo minimally invasive surgery (laparoscopic: 32.3% vs 26.3%; robotic: 20.0% vs 14.8%, p < 0.001), while patients without an ileostomy were more likely to have open surgery (32.7% vs 26.6%, p < 0.001). There were no statistically significant differences between patients with and without an ileostomy in rates of organ space SSI (7.0% vs 6.1%, p = 0.302), and AL (4.7% vs 3.5%, p = 0.064). There were also no statistically significant differences between patients with and without an ileostomy in rates of severe cases of AL (3.6% vs 2.7%, p = 0.177) or organ space SSI with reoperation (2.4% vs 1.5%, p = 0.069) (Table 3).

**Table 3:** Intraoperative characteristics and short-term postoperative outcomes for patients with and without an ileostomy

Variables (%)	Total (n = 4048)	No Ileostomy (n = 1244)	Ileostomy (n = 2804)	p-value
Preoperative chemotherapy	2016 (49.8)	446 (35.9)	1570 (56.0)	** < .001**
Preoperative radiation therapy	1845 (45.6)	403 (32.4)	1442 (51.4)	** < .001**
Operation time (Minutes)	294.48 (121.52)	274.11 (121.58)	303.51 (120.41)	** < .001**
Intraoperative transfusion	206 (5.1)	82 (6.7)	123 (4.4)	**0.002**
Operative approach				** < .001**
Open	1154 (28.5)	407 (32.7)	747 (26.6)	
Laparoscopy	1232 (30.4)	327 (26.3)	905 (32.3)	
Robotic	744 (18.4)	184 (14.8)	560 (20.0)	
Total length of stay (Days)	5.40 (11.70)	5.35 (13.14)	5.42 (11.00)	0.87
Reoperation	188 (4.6)	43 (3.5)	145 (5.2)	**0.017**
Organ space SSI	272 (6.7)	76 (6.1)	196 (7.0)	0.302
Organ space SSI w/ reoperation	87 (2.1)	19 (1.5)	68 (2.4)	0.069
Anastomotic leakage	176 (4.3)	43 (3.5)	133 (4.7)	0.064
Anastomotic leakage severity				0.177
Severe	133 (3.3)	33 (2.7)	100 (3.6)	
Mild	43 (1.1)	10 (0.8)	33 (1.2)	

Bold p-values are < 0.05, indicating statistically significant difference between the cohorts in comparison. Continuous variables and categorical variables were analyzed with univariate analysis and ANOVA tests respectively (proportions and standard deviations were reported in the bracket)

The rates of organ space SSI and AL in patients with and without an ileostomy were compared in each cohort separately (upper, middle, and lower rectal cancers). There were no statistically significant differences between patients with and without an ileostomy with low rectal cancer in rates of organ space SSI (6.0% vs 7.2%, p = 0.501), AL (4.4% vs 2.4%, p = 0.153), organ space SSI with reoperation (2.1% vs 1.6%, p = 0.626) or severe AL (3.0% vs 1.2%, p = 0.285). There were no statistically significant differences between patients with and without an ileostomy in the mid rectal cancer cohort in organ space SSI (7.5% vs 7.2%, p = 0.88), AL (5.0% vs 3.4%, p = 0.162), severe AL (3.7% vs 2.6%, p = 0.376), or organ space SSI with reoperation (2.3% vs 1.3%, p = 0.189). There were also not statistically significant differences between patients with and without an ileostomy in the upper rectal cancer cohort in AL (4.8% vs 4.0%, p = 0.538), severe AL (4.2% vs 3.4%, p = 0.806), or organ space SSI with reoperation (3.4% vs 1.7%, p = 0.078). Surprisingly, patients with an ileostomy in the upper rectal cancer cohort had significantly higher rate of organ space SSI (7.5% vs 4.6%, p = 0.049) (Table 4).

**Table 4:** Intraoperative characteristics and short-term postoperative outcomes subanalysis of patients with and without an ileostomy by different tumor locations

	Lower (n = 1166)			Middle (n = 1836)			Upper (n = 1046)		
Variables (%)	No Ileostomy (n = 251)	Ileostomy (n = 915)	p-value	No Ileostomy (n = 469)	Ileostomy (n = 1367)	p-value	No Ileostomy (n = 524)	Ileostomy (n = 522)	p-value
Preoperative chemotherapy	124 (49.4)	529 (57.8)	0.054	214 (45.6)	784 (57.4)	** < .001**	108 (20.6)	257 (49.2)	** < .001**
Preoperative radiation therapy	114 (45.4)	479 (52.3)	0.145	207 (44.1)	729 (53.3)	**0.003**	82 (15.6)	234 (44.8)	** < .001**
Operation Time	305.20 (140.98)	322.88 (126.59)	0.056	284.69 (123.99)	296.03 (118.86)	0.078	249.76 (103.50)	289.14 (108.82)	** < .001**
Intraoperative transfusion	21 (8.4)	43 (4.7)	**0.024**	34 (7.2)	54 (4.0)	**0.004**	28 (5.3)	26 (5.0)	0.791
Operative approach			** < .001**			**0.042**			**0**.**008**
Open	99 (39.4)	181 (19.8)		153 (32.6)	400 (29.3)		155 (29.6)	166 (31.8)	
Laparoscopy	49 (19.5)	301 (32.9)		117 (24.9)	420 (30.7)		161 (30.7)	184 (35.2)	
Robotic	42 (16.7)	231 (25.2)		81 (17.3)	257 (18.8)		61 (11.6)	72 (13.8)	
Total length of stay (Days)	4.49 (16.90)	5.64 (8.97)	0.147	6.59 (8.75)	5.48 (10.91)	**0**.**046**	4.66 (14.25)	4.87 (14.06)	0.813
Reoperation	11 (4.4)	47 (5.1)	0.626	16 (3.4)	71 (5.2)	0.117	16 (3.1)	27 (5.2)	0.084
Organ space SSI	18 (7.2)	55 (6.0)	0.501	34 (7.2)	102 (7.5)	0.88	24 (4.6)	39 (7.5)	**0**.**049**
Organ space SSI w/ reoperation	4 (1.6)	19 (2.1)	0.626	6 (1.3)	31 (2.3)	0.189	9 (1.7)	18 (3.4)	0.078
Anastomotic leakage	6 (2.4)	40 (4.4)	0.153	16 (3.4)	68 (5.0)	0.162	21 (4.0)	25 (4.8)	0.538
Anastomotic leakage severity			0.285			0.376			0.806
Severe	3 (1.2)	27 (3.0)		12 (2.6)	51 (3.7)		18 (3.4)	22 (4.2)	
Mild	3 (1.2)	13 (1.4)		4 (0.9)	17 (1.2)		3 (0.6)	3 (0.6)	

Bold p-values are < 0.05, indicating statistically significant difference between the cohorts in comparison. Continuous variables and categorical variables were analyzed with univariate analysis and ANOVA tests respectively (proportions and standard deviations were reported in the bracket)

As for the entire cohort, on multivariate analysis controlling for confounding variables of preoperative patient characteristics (age, sex, BMI, etc.), comorbidities (dyspnea, diabetes, etc.), preoperative chemotherapy and radiation therapy, intraoperative characteristics (operation time, operational approach, blood transfusion, etc.), adding an ileostomy did not decrease the odds of organ space SSI, AL, severe AL, or organ space SSI with reoperation (Supplement Table 1). When running a similar multivariate analysis for patients with upper, middle and lower rectal cancers, adding an ileostomy did not decrease the odds of space organ SSI, AL, organ space SSI with reoperation, or severe AL (Supplement Table 2a,b,c).

## Discussion

The data provided by the present case–control study on short-term outcomes following LAR for rectal cancer with and without an ileostomy were derived from a large national database. By comparing the outcomes both in the entire cohort and in sub-cohorts of different tumor locations, adding a diverting ileostomy did not improve the rates of postoperative AL or organ space SSI; either mild or severe cases that required a re-intervention or a reoperation. Such conclusions are supported by both descriptive statistical analysis and multivariable logistic regression analysis that controls for other variables.

Patients with lower and middle rectal cancers had higher rates of preoperative chemo- and radiation therapy, coinciding with higher rates of diverting ileostomy. Interestingly, patients with lower and middle rectal cancer had a lower chance of undergoing open surgery and a higher chance of undergoing robotic surgery (but not laparoscopic surgery). For the entire cohort, patients who had an ileostomy were more likely to undergo minimally invasive surgery (laparoscopic and robotic) and less likely to undergo open surgery.

The findings of the present case–control study agree with a few recent studies that similarly showed no significant benefits in short-term postoperative outcomes of adding an ileostomy to LAR for rectal cancer [4, 8, 9, 13]. A retrospective institutional study in 2020 by Niu et al. included 347 patients from 2011 to 2018 and showed that a protective ileostomy does not prevent anastomotic leakage after anterior resection for rectal cancer or the severity of the anastomotic leak [9]. The authors hypothesized that a protective stoma does not substantially improve local blood supply or nutrition, key contributing factors to AL, and may increase the incidence of other complications.

Munshi et al. in a 2023 retrospective clinical study using the Swedish Colorectal Cancer Registry (SCRCR) found that a de-functioning stoma does not help reduce AL and reoperation rates in rectal cancer patients undergoing anterior resection. This study compared 1,879 patients operated on from 2007 to 2009 to a cohort of 1809 patients from 2016 to 2018 and showed that although more recent patients had a 5% higher rate of getting a diverting ileostomy, there was no change in AL rates (9.2% vs 8.2%, p = 0.35). In the 2007–2009 cohort, 72.2% of patients received neoadjuvant therapy, 5.4% underwent laparoscopic surgery and 71.6% had a de-functioning ileostomy, while in the 2016–2018 cohort the rates were 63.2%, 58.8%, and 76.7%, respectively [8].

A prospective multi-center study of 2,729 rectal cancer patients conducted by Gastinger et al. between 2000 and 2001 also reported no significant decrease of the overall rate of AL for patients who had a protective stoma, although patients with a stoma had fewer leaks that required surgery [4]. The latter suggests that an ileostomy does not reduce AL leak rates but mostly minimizes the severity of AL.

The conclusions of the present case–control study disagree with several meta-analyses comparing postoperative surgical outcomes in rectal cancer patients undergoing LAR with and without an ileostomy [1–3, 5, 6, 10, 11]. A meta-analysis cited by the 2020 American Society of Colon and Rectal Surgeons’ clinical practice guidelines for rectal cancer management pooled various comparative studies and RCTs showing that a de-functioning ileostomy decreases AL incidence [11, 14]. A couple of other randomized controlled trials (RCT) similarly showed that having a de-functioning stoma significantly reduces AL incidence and postoperative outcomes. A large multicenter RCT by Matthiessen et al. included a total of 234 patients from 1999 to 2005 randomized by 21 hospitals and showed that patients without de-functioning stoma have a significantly higher leakage rate of 28% compared with 10.3% in patients with a stoma (p < 0.001) [7]. However, most of the studies included were older studies with relatively small numbers of patients. Additionally, these studies grouped all rectal cancer locations into one cohort and did not provide a sub-analysis of LAR with and without an ileostomy for different tumor locations.

The strengths of the present case–control study include the use of a large national database that is representative and prospectively collected in a consistent manner by dedicated clinical reviewers. Our study is also unique in the sub-analysis of effectiveness of a diverting ileostomy during LAR for different tumor locations. Variability was also minimized by including elective operations only. The limitations of the present study lie in its retrospective nature, and the relatively smaller cohort size for sub-analysis by tumor location. Additionally, potential selection bias cannot be ruled out, as the present study does not include data on the specific type of treatment rendered for AL. The classification of leak severity is limited to the definition by NSQIP. There are also coding inconsistencies expected in using large national databases such as NSQIP and possibility of type II errors [15]. Finally, the use of a large-scale database can lead to statistically significant results that may not be considered clinically relevant.

Although our study demonstrates that ileostomy does not significantly reduce the rate or severity of anastomotic leaks (AL), this finding does not negate the efficacy reported in existing studies. For certain cases, particularly when significant risk factors for AL are present—such as patient comorbidities, chemoradiation status, or the technical complexity of the operation—ileostomy has been shown to help mitigate AL severity according to previous research [7, 11, 14]. A diverting ileostomy is not without a cost; there are both short- and long-term stoma-related morbidities, including the risk for chronic kidney injury, permanent stoma, and higher risks of readmission. Additionally, stoma reversal is also associated with complications [9, 14]. As such, the authors of the present study believe that more stringent criteria based on high level recent studies should better serve to guide surgeons as to when to divert rectal cancer patients undergoing LAR. With recent advances in surgical techniques and minimally invasive approaches, the present study provides a necessary update on the current landscape in surgical practices for rectal cancer treatment using a large national database. The present study may further help guide surgeons and patients when planning surgery for removal of rectal tumor based on specific tumor location.

## Supplementary Information

Below is the link to the electronic supplementary material.

Supplementary file1 (DOCX 27 KB)

## Data Availability

The data used in this study are available from the American College of Surgeons National Surgical Quality Improvement Program (ACS NSQIP) database. Access to the database is restricted and requires approval from the ACS. Data can be made available upon reasonable request and with permission from the ACS NSQIP program.
