# Supplementary material for: Short-term outcomes of low anterior resection with and without ileostomy for low, mid and upper rectal cancers
Source: Updates Surg. 2025 Jan 23;77(3):629–36. doi: 10.1007/s13304-025-02088-2 (PMC12226602; doi:10.1007/s13304-025-02088-2)
Supplement: Supplementary file 1 — Supplementary file1 (DOCX 27 KB) [file 13304_2025_2088_MOESM1_ESM.docx]

Supplement Table 1: Multivariable logistic regression analysis of factors associated with AL, Organ Space SSI, and overall surgical complications in the entire cohort (-: removed by backward elimination)

|  | **Dependent Variables (entire cohort, n = 4048)** | | | | | | | | | | | |
| --- | --- | --- | --- | --- | --- | --- | --- | --- | --- | --- | --- | --- |
|  | **Anastomotic Leakage** | | | | **Organ Space SSI** | | | | **Overall Surgical Complications** | | | |
| **Independent Variables** | OR | 95% CI for OR | | p-value | OR | 95% CI for OR | | p-value | OR | 95% CI for OR | | p-value |
| **Sex** | 1.8 | 1.601 | 2.001 | **0.003** | 1.42 | 1.25 | 1.578 | **0.032** | - | | | |
| **BMI** | - | | | | - | | | | 1.03 | 1 | 1.054 | **0.004** |
| **Age** | - | | | | - | | | | - | | | |
| **ASA Classification** | - | | | | - | | | | - | | | |
| **Current Smoker** | - | | | | - | | | | 1.31 | 1.159 | 1.469 | 0.078 |
| **Weight Loss (>10%)** | - | | | | - | | | | 1.78 | 1.534 | 2.03 | **0.02** |
| **Dyspnea** | - | | | | - | | | | - | | | |
| **COPD** | - | | | | - | | | | - | | | |
| **Steroid** | - | | | | - | | | | - | | | |
| **Disseminated Cancer** | - | | | | - | | | | 1.44 | 1.242 | 1.642 | 0.068 |
| **Diabete** | - | | | | 1.42 | 1.23 | 1.606 | 0.067 | 1.36 | 1.205 | 1.519 | **0.049** |
| **Systemic Sepsis** | - | | | | - | | | | - | | | |
| **Hypertension** | - | | | | - | | | | - | | | |
| **Preoperative Chemotherapy** | - | | | | 0.71 | 0.49 | 0.928 | 0.116 | - | | | |
| **Preoperative Radiation Therapy** | - | | | | 1.22 | 1 | 1.44 | 0.365 | - | | | |
| **Operative Approach:** Lap | - | | | | Ref. |  |  | **0.019** | - | | | |
| Open | - | | | | 1.21 | 1.08 | 1.349 | 0.161 | - | | | |
| Robotic | - | | | | 0.72 | 0.54 | 0.886 | **0.05** | - | | | |
| Other | - | | | | 0.83 | 0.48 | 1.18 | 0.595 | - | | | |
| **Intraoperative Transfusion** | 3.42 | 3.167 | 3.673 | **<.001** | 0.33 | 0.1 | 0.549 | **<.001** | - | | | |
| **Operating Time** | - | | | | 1 | 1 | 1.002 | 0.084 | 1 | 1.001 | 1.001 | **0.012** |
| **Diverting Ileostomy** | - | | | | - | | | | - | | | |

Supplement Table 2a,b,c: Multivariable logistic regression analysis of factors associated with AL, Organ Space SSI, and overall surgical complications in each tumor location sub-cohort (-: removed by backward elimination)

|  | **Lower (n = 1166)** | | | | | | | | | | | |
| --- | --- | --- | --- | --- | --- | --- | --- | --- | --- | --- | --- | --- |
|  | **Anastomotic Leakage** | | | | **Organ Space SSI** | | | | **Overall Surgical Complications** | | | |
| **Independent Variables** | OR | 95% CI for OR | | p-value | OR | 95% CI for OR | | p-value | OR | 95% CI for OR | | p-value |
| **Sex** | 2.02 | 1.616 | 2.432 | 0.084 | - | | | | - | | | |
| **BMI** | - | | | | - | | | | 1.04 | 1.018 | 1.058 | 0.06 |
| **Age** | - | | | | - | | | | - | | | |
| **ASA Classification** | - | | | | - | | | | - | | | |
| **Current Smoker** | - | | | | - | | | | - | | | |
| **Weight Loss (>10%)** | - | | | | - | | | | - | | | |
| **Dyspnea** | - | | | | - | | | | - | | | |
| **COPD** | - | | | | - | | | | - | | | |
| **Steroid** | - | | | | - | | | | - | | | |
| **Disseminated Cancer** | 4.98 | 4.546 | 5.414 | **<.001** | 2.23 | 1.79 | 2.679 | 0.071 | - | | | |
| **Diabete** |  |  |  |  | - | | | | - | | | |
| **Systemic Sepsis** | 14.7 | 13.461 | 15.86 | **0.025** | - | | | | 8.43 | 7.364 | 9.498 | **0.046** |
| **Hypertension** | - | | | | - | | | | - | | | |
| **Preoperative Chemotherapy** | - | | | | 0.5 | 0.07 | 0.928 | 0.105 | - | | | |
| **Preoperative Radiation Therapy** | - | | | | 1.15 | 1.04 | 1.581 | 0.738 | - | | | |
| **Operative Approach:** Lap | - | | | | - | | | | - | | | |
| Open | - | | | | - | | | | - | | | |
| Robotic | - | | | | - | | | | - | | | |
| **Intraoperative Transfusion** | - | | | | - | | | | - | | | |
| **Operating Time** | - | | | | 1 | 1 | 1.003 | **0.025** | 1 | 1.001 | 1.003 | **0.026** |
| **Diverting Ileostomy** | - | | | | - | | | | 0.59 | 0.33 | 0.85 | **0.043** |

|  | **Middle (n = 1836)** | | | | | | | | | | | |
| --- | --- | --- | --- | --- | --- | --- | --- | --- | --- | --- | --- | --- |
|  | **Anastomotic Leakage** | | | | **Organ Space SSI** | | | | **Overall Surgical Complications** | | | |
| **Independent Variables** | OR | 95% CI for OR | | p-value | OR | 95% CI for OR | | p-value | OR | 95% CI for OR | | p-value |
| **Sex** | 1.92 | 1.619 | 2.213 | **0.028** | 1.88 | 1.64 | 2.115 | **0.008** | - | | | |
| **BMI** | - | | | | - | | | | - | | | |
| **Age** | - | | | | - | | | | - | | | |
| **ASA Classification** | - | | | | - | | | | - | | | |
| **Current Smoker** | - | | | | - | | | | - | | | |
| **Weight Loss (>10%)** | - | | | | - | | | | - | | | |
| **Dyspnea** | - | | | | - | | | | - | | | |
| **COPD** | - | | | | - | | | | - | | | |
| **Steroid** | - | | | | - | | | | - | | | |
| **Disseminated Cancer** | - | | | | - | | | | - | | | |
| **Diabete** | 1.76 | 1.448 | 2.08 | 0.073 | 1.92 | 1.67 | 2.174 | **0.01** | 1.74 | 1.522 | 1.95 | **0.01** |
| **Systemic Sepsis** | - | | | | - | | | | - | | | |
| **Hypertension** | - | | | | - | | | | - | | | |
| **Preoperative Chemotherapy** | - | | | | - | | | | - | | | |
| **Preoperative Radiation Therapy** | - | | | | - | | | | - | | | |
| **Operative Approach:** Lap | - | | | | - | | | | - | | | |
| Open | - | | | | - | | | | - | | | |
| Robotic | - | | | | - | | | | - | | | |
| **Intraoperative Transfusion** | 3.34 | 2.953 | 3.725 | **0.002** | 2.76 | 2.42 | 3.096 | **0.003** | - | | | |
| **Operating Time** | - | | | | - | | | | - | | | |
| **Diverting Ileostomy** | - | | | | - | | | | - | | | |

|  | **Upper (n = 1046)** | | | | | | | | | | | |
| --- | --- | --- | --- | --- | --- | --- | --- | --- | --- | --- | --- | --- |
|  | **Anastomotic Leakage** | | | | **Organ Space SSI** | | | | **Overall Surgical Complications** | | | |
| **Independent Variables** | OR | 95% CI for OR | | p-value | OR | 95% CI for OR | | p-value | OR | 95% CI for OR | | p-value |
| **Sex** | - | | | | - | | | | - | | | |
| **BMI** | - | | | | - | | | | 1.05 | 1.038 | 1.07 | **0.001** |
| **Age** | - | | | | - | | | | - | | | |
| **ASA Classification** | - | | | | - | | | | - | | | |
| **Current Smoker** | - | | | | - | | | | - | | | |
| **Weight Loss (>10%)** | - | | | | - | | | | 3.23 | 2.789 | 3.671 | **0.008** |
| **Dyspnea** | - | | | | - | | | | - | | | |
| **COPD** | - | | | | - | | | | - | | | |
| **Steroid** | - | | | | - | | | | - | | | |
| **Disseminated Cancer** | - | | | | - | | | | - | | | |
| **Diabete** | - | | | | - | | | | - | | | |
| **Systemic Sepsis** | - | | | | - | | | | - | | | |
| **Hypertension** | - | | | | - | | | | - | | | |
| **Preoperative Chemotherapy** | - | | | | - | | | | - | | | |
| **Preoperative Radiation Therapy** | - | | | | - | | | | - | | | |
| **Operative Approach:** Lap | Reference | |  | 0.059 | - | | | | Reference | |  | **0.028** |
| Open | 0.93 | 0.552 | 1.316 | 0.859 | - | | | | 1.98 | 1.702 | 2.254 | **0.013** |
| Robotic | 0.35 | -0.41 | 1.118 | 0.174 | - | | | | 0.74 | 0.292 | 1.196 | 0.514 |
| **Intraoperative Transfusion** | 5.21 | 4.767 | 5.653 | **<.001** | 4.85 | 4.47 | 5.233 | **<.001** | - | | | |
| **Operating Time** | - | | | | - | | | | - | | | |
| **Diverting Ileostomy** | - | | | | - | | | | - | | | |
